# Impairment of MLH1 and CDKN2A in oncogenesis of laryngeal cancer

**DOI:** 10.1038/sj.bjc.6601679

**Published:** 2004-04-06

**Authors:** M M Sasiadek, A Stembalska-Kozlowska, R Smigiel, D Ramsey, T Kayademir, N Blin

**Affiliations:** 1 Department of Genetics, Medical University, ul. Marcinkowskiego 1, Wroclaw 50-368, Poland; 2 Department of Pathophysiology, Medical University, ul. Marcinkowskiego 1, Wroclaw 50-368, Poland; 3 Institute of Mathematics, Technical University, Wroclaw, Poland; 4 Division of Molecular Genetics, Eberhard Karls University, Tübingen, Germany

**Keywords:** LSCC, *CDKN2A* (p16), *MLH1*, *RB1*

## Abstract

Our study aimed at elucidating which genetic alterations tend to form a network and could be applied as molecular markers of larynx squamous cell carcinoma (LSCC). A panel of genes involved in tumorigenesis was investigated. To search for the possible mechanisms of gene silencing, loss of heterozygosity (LOH) was analysed followed by testing DNA methylation and protein expression for those genes found with the highest frequency of LOH (*CDKN2A* (55.4%), *MLH1* (46.0%), *RB1* (35.7%)). A correlation of both LOH and hypermethylation with the loss of expression for *CDKN2A* and *MLH1* was found. Disrupted Rb pathway (loss of expression of *RB1* and/or of *CDKN2A*) in 55.9% of analysed cases confirmed the hypothesis that RB1 pathway is altered in head and neck squamous cell carcinomas, with *CDKN2A* (45%), rather than *RB1* (11.8%) being more frequently inactivated. In LSCC, LOH tends to occur together in gene pairs or triplets. The pair *MLH1*/*CDKN2A* and triplets *MLH1/TSG on 8p22/CDKN2A* and *MLH1/CDKN2A/RB1* are related to staging and grading. LOH in *MLH1* correlates with lower and LOH in *CDKN2A* with higher grades of LSCC. It can be concluded that *MLH1* and *CDKN2A* play an important role in LSCC development and progression.

Squamous cell carcinomas of the head and neck (HNSCC) are frequently occurring cancers with a mean incidence of around 5% of all cancers diagnosed worldwide annually. Despite intense efforts to develop early detection methods, as well as effective therapy in HNSCC, the overall 5-year survival rate of 50% remains among the lowest for the major cancers (Papadimitrakopoulou, 2000; Crowe *et al*, 2002). In the aetiology of HNSSC, both environmental and genetic factors are involved. Here, not only the cancer genes such as oncogenes, tumour suppressor genes (TSGs), mutator genes but also genes that modulate the individual susceptibility to environmental carcinogens, for example, genes involved in xenobiotic metabolism and DNA repair are involved in HNSCC development (Sturgis and Wei, 2002). This makes the genetics of HNSCC extremely complex (Fan, 2001). The situation becomes increasingly complicated since the definition of HNSCC covers a variety of tumours, which differ both in terms of their location within the upper aerodigestive tract and also in histopathology. Successive publications revealed the involvement of a variety of genes in the development and progression of HNSCC, such as oncogenes, for example, *MYC*, *RAS*, *ERBB2*, *BCL2*, *INT2* and TSG, for example, *TP53*, *CDKN2* (Voravud *et al*, 1997). It was also shown that a poor clinical course of HNSCC is linked to loss of heterozygosity (LOH) in multiple loci (Gleich *et al*, 1999), especially to allelic loss at 3p, 8q, 13q and 17p (Li *et al*, 1994; Scholnick *et al*, 1996; El-Naggar *et al*, 1997), while LOH at 9p21 and 17q31 is related to a high incidence of recurrent tumours (Matsuura *et al*, 1998). The karyotypes in HNSCC were shown to be complex with breakpoints underlying chromosomal alterations located mainly at 1p, 1q, 3p, 3q, 4q, 8p, 8q, 9p, 10p, 10q, 11q, 13p, 14p, 15p and 15q. Nonrandom patterns of chromosomal aberrations in the progression of HNSCC have been suggested (Jin *et al*, 1990; Van Dyke *et al*, 1994; Soder *et al*, 1995). Isochromosomes 8q, deletion 3p and homogenously staining regions at 11q13 were most often observed among the recurrent structural chromosomal aberrations (Van Dyke *et al*, 1994; Soder *et al*, 1995; Bergamo *et al*, 2000). The application of comparative genomic hybridisation (CGH) permitted the identification of chromosomal imbalances (Kujawski *et al*, 1999; Bergamo *et al*, 2000). The gains were observed mainly in 3q and 8q, while losses occurred in 3p and 22q. Also, a correlation between some clinical data and chromosomal alterations was reported, such as association between the gains of 1q and 2q and a refractory clinical response (Bergamo *et al*, 2000), and more frequent losses at 13q, 8p and 9q in metastatic than in primary tumours (Kujawski *et al*, 1999).

Although abundant molecular and cytogenetic data on HNSCC have been collected, neither critical genes nor a generally accepted genetic model of HNSCC development and progression have been described. The first model suggested for HNSCC combines, as an early event, multiple LOH with allelic loss at 9p, 3p, 17q, 4q and 13q. LOH at 18q and 8p are considered late genetic events (Califano *et al*, 1996). On the basis of already published cytogentic data, two main genetic pathways in HNSCC were suggested (Höglund *et al*, 2001) by using the two principal components of genomic imbalance (gains and losses): one with −1p, −1q and −7q as an early event followed by −8p and −4p, and another starting with +7q, and subsequently followed by +11pq, +8q and +1pq. Both then converge to a common set of imbalances: −3p, −9p and −11q. In the recently published ‘oncogenic trees’ for HNSCC progression, three subsets of tumours, which differed in their localisation (pharyngeal, laryngeal and oral squamous cell carcinoma), were analysed separately by using CGH (Huang *et al*, 2002). The authors proposed that generally, +3q/−3p is the most important chromosomal event in the genetic aetiology of HNSCC, which then may be followed by other chromosomal imbalances, occurring with various frequencies depending on cancer location.

Despite the lack of precise genetic information, it seems clear that HNSCC results from the accumulation of changes in genes controlling genomic stability, proliferation, apoptosis and invasion, and that these changes tend to form a functional network. Therefore, we decided to investigate the interconnections of genes involved in different pathways, which were observed to be most often altered in cancers. The rationale underlying the choice of genes to be tested was based on recently published results. Our collection consisted of tumour suppressors such as *HPC1, APC*, unknown TSG on 8p22*, TP53*, *TFF1*, *TFF2*, cell cycle controlling (*RB1, CDKN2A*) and mismatch repair genes (*MLH1, MSH2*), as well as ‘metastasis’ genes *NME1, NME2* and *NME3* (Scholnick *et al*, 1996; Gallo *et al*, 1997; Seifert *et al*, 1997; Matsuura *et al*, 1998; Ransom *et al*, 1998; Grati *et al*, 2000; Oba *et al*, 2001; Carvalho *et al*, 2002; Tsuda *et al*, 2002).

To search for possible changes leading to gene silencing, we started with LOH analysis. Then we evaluated the methylation status and finally the protein expression for genes showing the highest frequency of LOH. All genetic analyses were performed with a homogenous set of 62 larynx squamous cell carcinoma (LSCC), which represents the most common type of HNSCC.

The aim of our study was to establish which of the analysed genes are likely to be critical in LSCC and which genetic alterations tend to occur together to form a network of molecular events and then could be applied as molecular markers of clinical outcome.

## MATERIALS AND METHODS

### Samples

To analyse the homogeneous set of primary LSCC, cancer tissues and matched blood samples were obtained from 62 patients diagnosed with the primary LSCC in the Department of Otolaryngology, Wroclaw Medical University, Poland. All the tumours were diagnosed histopathologically according to the World Health Organisation's criteria as carcinoma planoepitheliale keratodes (33 cases), akeratodes (25 cases) and keratoblasticum (four cases). Biological material for molecular analysis was collected before chemotherapy and/or radiotherapy. Cancer tissues for molecular analyses were dissected from the central part of tumour. None of the patients had a history of hereditary cancer. A clinicohistopathological characterisation of patients is summarised in Table 1

**Table 1:** Clinicohistopathological characteristics of group of patients with LSCC

**Age**	**Sex**	**Histopathology**	**Grading**	**T**	**N**	**M**	**Staging**
59	M	k	2	3	2	0	4a
48	F	k	3	4	0	0	4a
54	M	k	2	4	0	0	4a
54	M	k	2	3	2	0	4a
57	M	k	2	4	0	0	4a
59	M	k	1	4	0	0	4a
64	M	k	2	4	1	0	4a
50	M	ak	2	3	0	0	3
62	M	k	1	4	1	0	4a
66	M	k	2	4	0	0	4a
65	M	ak	2	3	0	0	3
45	M	ak	2	4	1	0	4a
61	M	k	1	1	0	0	1
63	M	k	2	3	0	0	3
60	M	k	3	4	0	0	4a
67	M	k	2	3	0	0	3
62	M	k	2	3	0	0	3
64	M	ak	1	3	0	0	3
56	M	ak	3	3	0	0	3
68	M	ak	3	4	3	0	4b
54	M	ak	2	4	0	0	4a
71	M	kb	2	4	1	0	4a
60	M	k	3	3	0	0	3
50	F	ak	2	4	0	0	4a
59	M	k	1	3	0	0	3
69	M	ak	3	1	0	0	1
51	M	k	2	3	0	0	3
44	M	k	2	2	0	0	2
57	M	ak	2	3	0	0	3
61	M	ak	3	3	0	0	3
48	M	kb	2	3	1	0	3
61	M	ak	3	3	2	0	4a
69	F	ak	3	4	2	0	4a
56	M	ak	2	3	2	0	4a
43	M	ak	2	3	0	0	3
50	M	ak	2	3	0	0	3
67	M	kb	2	3	0	0	3
58	M	ak	3	3	0	0	3
63	M	kb	2	4	0	0	4a
71	M	ak	3	1	0	0	1
60	M	k	2	1	0	0	1
71	M	k	2	4	0	0	4a
50	M	k	1	3	0	0	3
49	M	k	1	2	0	0	2
69	M	ak	3	4	0	0	4a
61	M	k	2	3	1	0	4a
60	M	ak	2	3	0	0	3
49	M	ak	1	4	1	0	4a
61	F	k	3	3	0	0	3
51	M	ak	3	4	2	0	4a
60	M	ak	3	3	0	0	3
46	M	ak	2	3	0	0	3
57	F	ak	2	2	0	0	2
48	M	k	2	3	0	0	3
51	M	k	2	4	0	0	4a
61	M	k	1	3	0	0	3
64	M	k	2	4	1	0	4a
51	M	k	2	4	2	0	4a
49	M	k	1	4	0	0	4a
55	M	k	1	4	0	0	4a
52	M	k	1	4	0	0	4a
56	F	k	2	4	0	0	4a

F=female; M=male; k=carcinoma planoepitheliale keratodes; ak=carcinoma planoepitheliale akeratodes; kb=carcinoma planoepitheliale keratoblasticum; LSCC=larynx squamous cell carcinoma.

.

### DNA amplification and LOH analysis

DNA was isolated from cancer tissue and corresponding peripheral blood lymphocytes following standard procedures. Extracted DNA (3 *μ*g) was DOP-amplified (Degenerate Oligonucleotide Primed-PCR^2^ with the NRich-kit, Genpak, UK) following the manufacturer's guidelines. To test for genes important in carcinogenesis, a panel of microsatellite markers was applied: D1S2883, D2S123, D3S1611, D5S346, D8S254, *NME1, NME2, NME3*, *TP53, TFF1, TFF2*, D9S171 and *RB1*. The sequences for all primers are listed in the Genome Database (http//gdbwww.gdb.org). The LOH was studied in larynx carcinoma DNA and in matched constitutional DNA from the original series. PCR was performed according to the standard protocol in a PTC-200 thermocycler (MJ Research, USA). Fluorescent PCR products were pooled and resolved on a 4% polyacrylamide gel supplemented with 7 M urea in an ABI-377 sequencing device. Cold PCR amplification of TFF1 and TFF2 was performed as described previously (Carvalho *et al*, 2002). Sizes and quantity of marker alleles were evaluated by a semiautomated analysis using GENESCAN software, version 3.1, and the GENOTYPER software package, version 2.0 (Applied Biosystems, USA). Allelic loss was defined as a more than 70% reduction in the tumour peak area compared to the peak area of corresponding normal tissue (Karnik *et al*, 1998).

PCR with methylation-specific primers (MSP-PCR) was used to assess the promoter methylation of the CDKN2A. Genomic DNA was bisulphite treated followed by amplification performed by cold PCR as described previously using the following primers: forward, 5′-attagtggagattattgttttaga-3′; reverse, 5′-aaaaaaaacataccttacctatct-3′ (Herman *et al*, 1996). Cycling conditions were 30 s at 94°C, 30 s at 55°C and 30 s at 72°C for 35 cycles. Methylation of the *MLH1* promoter regions was examined using previously described digestion protocols with some modifications. Normal and tumour DNA were digested using *Hpa*II and *Msp*I enzymes. The *MLH1* promoter region was analysed using two pairs of specific primers: MLH1-881 and MLH1-1219 or MLH1-881 and MLH1-1470 described elsewhere. (Papadimitrakopoulou, 2000; Rainho *et al*, 2001; Fiedler *et al*, 2002) PCR products were separated in nondenaturated 6% PAA gels, stained in ethidium bromide and directly visualised by UV illumination.

### Immunostaining

Immunostaining for MLH1, P16 and RB was performed using the streptovidin–biotin peroxidase method according to standard procedures. The samples were immunostained using the following monoclonal antibodies: MLH1 IgG_1_ (clone G168-15, BD Biosciences, Germany), P16 IgG_2a_ (clone F-12, sc-1661, Santa Cruz Biotechnology Inc., USA) and RB IgG_1_ (clone RB1 1F8, Dako, Denmark) in the dilution 1 : 400, 1 : 50 and 1 : 100, respectively. Specimens were counterstained with haematoxylin and analysed in a light microscope. Normal human laryngeal tissue was used as a negative control. The number of positive cells per high field was assessed. The immunoreactivity results were recorded as positive when at least 20% of nuclear cells were stained positively, and any reduction below 20% in the number of stained cells was considered as an abnormal pattern.

### Statistical analysis

The Spearman's correlation coefficient was used to analyse associations between LOH at two loci. In order to find associations between LOH at three loci, a data mining method was applied (Mannila, 1997). First, we found triplets of loci such that LOH occurred at all three loci often. Such triplets were said to be associated if for each of the three possible pairings of loci in the triplet, the following condition is satisfied: the probability of LOH occurring at the locus where LOH was less common, given that LOH occurred at the other locus, was above a given threshold. The threshold chosen ensured that LOH at any of the three loci is positively correlated with LOH at the remaining loci for all the associated triplets. In order to investigate the effect of the explanatory variables on the clinical variables observed, the Mann–Whitney rank test was used. In addition, Student's *t*-tests and ANOVA tests were used to test the effect of the explanatory variables on grading, which had a bell-shaped distribution.

## RESULTS

The LOH analysis was performed by using 13 polymorphic microsatellite markers, on the DNA isolated from the 62 LSCC and corresponding normal tissue. All cases were informative (heterozygous) in at least 60% of analysed loci. Loss of heterozygosity was most frequently observed in the loci linked to the genes: *CDKN2A* (55.4%), *MLH1* (46.0%), TSG on 8p22 (38%), *RB1* (35.7%) and *NME1* (21%). According to the definition that LOH can be accepted as specific if occurring in given loci in no less than 20% of analysed cases (Ah-See *et al*, 1994; Li *et al*, 1994; Nawroz *et al*, 1994), the above-mentioned genes were chosen for further studies with the exception of metastasis-related gene *NME1*, because in the analysed group of patients no distant metastases were observed. Further, molecular analysis was not performed for TSG on 8p22 because the critical gene in this region is not yet specified (Fujiwara *et al*, 1995; Scholnick *et al*, 1996).

The analysis of the *CDKN2A* gene showed altered methylation in 37.5% and a decrease of protein expression in 45% of cases. For the *MLH1* gene, altered methylation was observed in 22.6% and downexpression in 27.5% of cases. For the *RB1* gene, decrease in protein expression was noted only in 11.8% of cases (seven out of 59); therefore, the promoter methylation status was not evaluated. The results are summarised in Table 2

**Table 2:** Results of analysis of LOH, methylation and protein expression in LSCC

		**LOH^b^**		**Aberrant methylation**		**Decreased expression**	
**Gene**	**Chromosomal localisation**	**No. of cases**	**(%)**	**No. of cases**	**(%)**	**No. of cases**	**(%)**
*MLH1*	3p22	23/50	46.0	14/62	22.6	14/51	27.5
*TSG*^c^	8p22	21/55	38.0				
*RB1*	13q14	20/56	35.7			7/59	11.8
*CDKN2A HPC1*	9p21	31/56	55.4	21/56	37.5	27/60	45
*APC*	1q24	6/44	13.6				
*MSH2*	5q21	10/60	16.6				
*TP53*	2p16	7/49	14.2				
*NME1*	17p13	4/51	7.8				
*NME2*	17q21	12/57	21.0				
*NME3*	17q21	4/43	9.3				
*TFF1*	17q21	7/54	12.9				
*TFF2*	21q22.3	2/19	10.5				

a The percentage was calculated for the informative cases.

b LOH,loss of heterozygosity; LSCC=larynx squamous cell carcinoma.

c TSG (unknown tumour suppressor gene on 8p22).

. The analysis of correlation of both LOH and aberrant methylation with the loss of protein expression for *CDKN2A* and *MLH1* genes showed a significant value (*P*<0.01, Spearman's test). However, no statistical significance was discernible between the LOH and loss of protein expression for the *RB1* locus. The following correlations of LOH, methylation and loss of protein expression with tumour grading were observed: negative for *MLH1* and positive for *CDKN2A* (Table 3

**Table 3:** Results of analysis of LOH, methylation and protein expression of CDKN2A and MLH1 in LSCC

	**CDKN2A**			**MLH1**		
**Grading^(G)^**	**LOH^b^ %**	**Aberrant methylation^c^ %**	**Decreased expression^d^ %**	**LOH^b^ %**	**Aberrant methylation^c^ %**	**Decreased expression^c^ %**
1	45.5	27.27	50	62.5	33.33	41
2	47.05	32.25	33.33	50	27.7	26.9
3	83.33	57.14	66.66	30	6.6	0

a The percentage was calculated for the informative cases.

b LOH, loss of heterozygosity; LSCC=larynx squamous cell carcinoma.

c Statistically significant results – *P*<0.01; ANOVA.

d Statistically significant results – *P*<0.05; ANOVA.

).

To search for genetic alterations that tend to occur together to form a network of molecular events, Spearman's test and association analysis were applied. We noted that LOH in the following genes tends to occur in pairs: *TSG on 8p22*/*NM*E1, *MLH1*/*CDKN2A* (*P*<0.01, Spearman's test) and with lower statistical significance in *TSG on 8p22*/*MLH2, TSG on 8p2*/*NME3*, *MSH2*/*NME2*, *MSH2*/*APC* (*P*<0.05, Spearman's test; Table 4

**Table 4:** Pairs and triplets of genes in which LOH tends to occur together in LSCC.

**Pairs**	
*TSG*^a^/*NME1*	*P*<0.01 (Spearman's test)
*MLH1 /CDKN2A*	
	
*TSG*/*MSH2*	*P*<0.05 (Spearman's test)
*TSG*/*NME3*	
*MSH2/NME2*	
*MSH2/APC*	
	
Triplets	
*MLH1/CDKN2A/TSG*	Association analysis
*MLH1/CDKN2A/RB*	

a *TSG* – unknown tumour suppressor gene on 8p22. LSCC=larynx squamous cell carcinoma.

). Analysis of the LOH pairs mentioned above and clinicohistopathological features of disease showed a positive correlation for only one pair: *MLH1*/*CDKN2A* and staging (*P*<0.05, ANOVA) and grading (*P*<0.01, ANOVA). For this pair, LOH in *MLH1* correlates negatively but LOH in *CDKN2A* correlates positively with grading (*P*<0.01, ANOVA). Further application of association analysis for triple parameters indicated a correlation for the following sets: *MLH1/CDKN2A/TSG on 8p22* and *MLH1/CDKN2A/RB1* (Table 4). Detailed statistical analysis showed that in both triplets, LOH in *MLH1* correlates with lower, and in *CDKN2A* with higher grading (*P*<0.01, ANOVA), but LOH in *TSG on 8p22* and *RB1* gene are not directly linked to tumour grading.

## DISCUSSION

Analysis of allelic loss (LOH) is widely applied in searching for tumour suppressor genes involved in the process of neoplastic transformation. The analysis of LOH indicated the involvement of a variety of genes in the development and progression of LSCC (Rainho *et al*, 2001; dos Reis *et al*, 2002; Fiedler *et al*, 2002; Gunduz *et al*, 2002). It is hypothesised that LSCC develops after the accumulation of six to 10 independent genetic events (Renan, 1993). Therefore, our study focused on searching for alterations in a chosen panel of genes reported to be frequently altered in manifold cancers. In our series of LSCCs, LOH was most frequently observed in microsatellites linked to the following genes: *CDKN2A* (55.4%), *MLH1* (46.0%), *TSG on 8p22* (38%), *RB1* (35.7%) and *NME1* (21%). The function of some of them in tumorigenesis is well known. *CDKN2A* and *RB1* play an important role in the cell cycle control (in RB pathway) (Sherr, 1996; Yokoyama *et al*, 1996). *MLH1* belongs to the group of genes controlling mismatch repair (Deng *et al*, 1999; Wheeler *et al*, 1999; Arzimanoglou *et al*, 2002). However, the location of putative genes on 8p and their association with the development and progression of HNSCC are still disputed (Li *et al*, 1994; Nawroz *et al*, 1994; Fujiwara *et al*, 1995).

Therefore, we chose *CDKN2A*, *RB1* and *MLH1* for a more detailed molecular analysis. Since promoter methylation following LOH is frequently involved in the silencing of *CDKN2A* and *MLH1* (El-Naggar *et al*, 1997; Deng *et al*, 1999; Wheeler *et al*, 1999), but not *RB1* (Yokoyama *et al*, 1996; Gleich *et al*, 1999), the analysis of methylation of *CDKN2A* and *MLH1* was also performed. The positive correlation of both LOH and hypermethylation with loss of protein expression for *CDKN2A* and *MLH1* genes (*P*<0.01, Spearman's test) confirmed the thesis that these are the most important mechanisms for silencing the *CDKN2A* and *MLH1* genes (El-Naggar *et al*, 1997; Deng *et al*, 1999; Wheeler *et al*, 1999). Our results, showing impaired Rb pathway (loss of expression of *RB1* and/or of *CDKN2A*) in 55.9% of analysed cases confirmed the hypothesis that RB1 pathway (55.9%) is commonly disrupted in HNSCC development and progression, with *CDKN2A* (45%), rather than *RB1* (11.8%) being the frequent direct target for inactivation (Lang *et al*, 2002). Despite the fact that in our series downregulation of *MLH1* was observed in 27.5% of cases, analysis of microsatellite instability (MSI) by using BAT 25, BAT 26 and BAT 40 markers showed only low-frequency MSI (MSI-L) in three out of 62 analysed cases (published elsewhere) (Sasiadek *et al*, 2002). Therefore, we concluded that in LSCC inactivation of MLH1 does not lead to MSI, in contrast to the observation for hereditary nonpolyposis colon cancer (Wheeler *et al*, 1999). A similar observation was reported by Arzimanoglou *et al*. (2002), who observed frequent LOH at *MLH1* and negligible DNA instability in ovarian cancer. These results support the hypothesis that microsatellite stability is controlled by a variety of genes (Giannini *et al*, 2002). Statistical analysis of LOH in *MLH1, CDKN2A* and *RB1* genes and clinicohistopathological features of the disease disclosed that LOH in *MLH1* and *CDKN2A* correlates only with tumour grading. Our results suggest that LOH in *MLH1* is characteristic for lower, while LOH in *CDKN2A* occurs in higher grades of LSCC (Table 3). We searched for the significance of combinations of LOH in two or three loci taking into account the opinion of Huang *et al* (2002) that sole analysis of single genetic alterations may neglect the important role of a combination of two or more alterations during the progression of cancer. We found six pairs and two triplets of genes in which LOH tends to occur together. The analysis of their correlation with clinicohistopathological features of the disease proved that one pair (*MLH1/CDKN2A)* and both triplets are related to staging and grading. We observed that in each of these cases LOH in *MLH1* correlates with lower and LOH in *CDKN2A* with higher grades of LSCC. Similar correlations were observed in the analysis of LOH in single loci. Therefore, it can be postulated that *MLH1* and *CDKN2A* play an important role in LSCC development and progression.

## References

[bib1] Ah-See K, Cooke TG, Pickford IR, Soutar D, Balmain A (1994) An allelotype of squamous carcinoma of the head and neck using microsatellite markers. Cancer Res 54: 1617–1621 8137268

[bib2] Arzimanoglou II, Hansen LL, Chong D, Li Z, Psaroudi MC, Dimitrakakis C, Jacovina AT, Shevchuk M, Reid L, Hajjar KA, Vassilaros S, Michalas S, Gilbert F, Chervenak FA, Barber HR (2002) Frequent LOH at hMLH1, a highly variable SNP in hMSH3, and negligible coding instability in ovarian cancer. Anticancer Res 22: 969–975 12014680

[bib3] Bergamo NA, Rogatto SR, Poli-Federico RC, Reis PP, Kowalski LP, Yielenska M, Squire A (2000) Comparative genomic hybridization analysis detects frequent over-representation of DNA sequences at 3q, 7p and 8q in head and neck carcinomas. Cancer Genet Cytogenet 119: 48–55 10812171 10.1016/s0165-4608(99)00213-7

[bib4] Califano J, van der Riet P, Westra W, Nawroz H, Clayman G, Piantadosi S, Corio R, Lee D, Greenberg B, Koch W, Sidransky D (1996) Genetic progression model for head and neck cancer: implications for field cancerization. Cancer Res 56: 2488–2492 8653682

[bib5] Carvalho R, Kayademir T, Soares P, Canedo P, Sousa S, Oliveira C, Leistenschneider P, Carneiro F, Gött P, Blin N, Seruca R, Machado JC (2002) LOH and promoter methylation, but not mutation, may underlie loss of TFF1 in gastric carcinoma. Lab Invest 82: 1319–1326 12379766 10.1097/01.lab.0000029205.76632.a8

[bib6] Crowe DL, Hacia JG, Hsieh C-L, Sinha UK, Rice DH (2002) Molecular pathology of head and neck cancer. Histol Histopathol 17: 909–914 12168802 10.14670/HH-17.909

[bib7] Deng G, Chen A, Hong J, Chae HS, Kim YS (1999) Methylation of CpG in a small region of the hMLH1 promoter invariably correlates with the absence of gene expression. Cancer Res 59: 2029–2033 10232580

[bib8] dos Reis PP, Poli-Federico RC, dos Santos RM, Nishimoto IN, Kowalski LP, Rogatto SR (2002) Distinct regions of loss of heterozygosity on 22q in different sites of head and neck squamous cell carcinomas. Med Sci Monit 8: BR89–BR94 11889455

[bib9] El-Naggar AK, Lai S, Clyman G, Lee JKJ, Luna MA, Goepfert H, Batsakis JG (1997) Methylation, a major mechanism of p16/CDKN2 gene inactivation in head and neck squamous carcinoma. Am J Path 151: 1767–1773 9403727 PMC1858347

[bib10] Fan CY (2001) Genetic alterations in head and neck cancer: interactions among environmental carcinogens, cell cycle control, and host DNA repair. Curr Oncol Rep 3: 66–71 11123872 10.1007/s11912-001-0045-0

[bib11] Fiedler W, Hoppe C, Schimmel B, Koscielny S, Dahse R, Bereczki Y, Claussen U, Ernst G, von Eggeling F (2002) Molecular characterization of head and neck tumors by analysis of telomerase activity and a panel of microsatellite markers. Int J Mol Med 9: 417–423 11891539

[bib12] Fujiwara Y, Ohata H, Kuroki T, Koyama K, Tsuchiya E, Monden M, Nakamura Y (1995) Isolation of a candidate tumor suppressor gene on chromosome 8p21.3-p22 that is homologous to an extracellular domain of the PDGF receptor beta gene. Oncogene 10: 891–895 7898930

[bib13] Gallo O, Franchi A, Chiarelli I, Porfirio B, Grande A, Simonetti L, Bocciolini C, Fini-Storchi O (1997) Potential biomarkers in predicting progression of epithelial hyperplastic lesions of the larynx. Acta Otolaryngol Suppl 527: 30–38 9197477 10.3109/00016489709124030

[bib14] Giannini G, Ristori E, Cerignoli F, Rinaldi C, Zani M, Viel A, Ottin L, Crescenzi M, Martinotti S, Bignami M, Frati L, Screpanti I (2002) Human MRE11 is inactivated in mismatch repair-deficient cancers. EMBO Rep 3: 248–254 11850399 10.1093/embo-reports/kvf044 PMC1084012

[bib15] Gleich LL, Li YQ, Wang X, Stambrook PJ, Gluckman JL (1999) Variable genetic alterations and survival in head and neck cancer. Head Neck Surg 125: 949–952 10.1001/archotol.125.9.949 10488977

[bib16] Grati FR, Sirchia SM, Garagiola, Sironi E, Galioto S, Rossella F, Serafini P, Dulcetti F, Bozzetti A, Brusati R, Simoni G (2000) Losses of heterozygosity in oral and oropharyngeal epithelial carcinomas. Cancer Genet Cytogenet 118: 57–61 10731592 10.1016/s0165-4608(99)00199-5

[bib17] Gunduz M, Ouchida M, Fukushima K, Ito S, Jitsumori Y, Nakashima T, Nagai N, Nishizaki K, Shimizu K (2002) Allelic loss and reduced expression of the ING3, a candidate tumor suppressor gene at 7q31, in human head and neck cancers. Oncogene 21: 4462–4470 12080476 10.1038/sj.onc.1205540

[bib18] Herman JG, Graff JR, Myohanen S, Nelkin BD, Baylin SB (1996) Methylation-specific PCR: a novel PCR assay for methylation status of CpG islands. Proc Natl Acad Sci USA 93: 9821–9826 8790415 10.1073/pnas.93.18.9821 PMC38513

[bib19] Höglund M, Gisselsson D, Mandahl N, Johansson B, Mertens F, Mitelman F, Säll T (2001) Multivariate analyses of genomic imbalances in solid tumors reveal distinct and converging pathways of karyotypic evolution. Genes Chromosomes Cancer 31: 156–171 11319803 10.1002/gcc.1129

[bib20] Huang Q, Yu GP, McCormick SA, Mo J, Datta B, Mahimkar M, Lazarus P, Schäffer AA, Desper R, Schantz SP (2002) Genetic differences detected by comparative genomic hybridisation in head and neck squamous cell carcinomas from different tumor sites: construction of oncogenetic trees for tumor progression. Genes Chromosomes Cancer 34: 224–233 11979556 10.1002/gcc.10062

[bib21] Jin Y, Higashi K, Mandahi N, Heim S, Wennerberg J, Biorklund A, Dictor M, Mitelman F (1990) Frequent rearrangement of chromosomal bands 1p22 and 11q12 in squamous cell carcinomas of head and neck. Genes Chromosomes Cancer 2: 198–204 2078510 10.1002/gcc.2870020306

[bib22] Karnik P, Paris M, Williams BRG, Casey G, Crowe J, Chen P (1998) Two distinct tumor suppressor loci within chromosome 11p15 implicated in breast cancer progression and metastasis. Hum Mol Genet 7: 895–903 9536095 10.1093/hmg/7.5.895

[bib23] Kujawski M, Sariomo-Rikala M, Gabriel A, Szyfter K, Knuutila S (1999) Recurrent DNA copy number losses associated with metastasis of larynx carcinoma. Genes Chromosomes Cancer 26: 253–257 10502324 10.1002/(sici)1098-2264(199911)26:3<253::aid-gcc10>3.0.co;2-a

[bib24] Lang JC, Borchers J, Danahey D, Smith S, Stover DG, Agrawal A, Malone JP, Schuller DE, Weghorst CM, Holinga AJ, Lingam K, Patel CR, Esham B (2002) Mutational status of overexpressed p16 in head and neck cancer: evidence for germline mutation of p16/p14ARF. Int J Oncol 21: 401–408 12118338 10.3892/ijo.21.2.401

[bib25] Li X, Lee NK, Ye Y-W, Waber PG, Schweizer C, Cheng Q-C, Nisen PD (1994) Allelic loss at chromosomes 3p, 8p, 13q and 17p associated with poor prognosis in head and neck cancer. J Natl Cancer Inst 86: 1524–1529 7932807 10.1093/jnci/86.20.1524

[bib26] Mannila (1997) Methods and problems in data mining. Afrati F, Kolaitis P (eds) The Proceedings of the International Conference on Database Theory, Delphi, Greece: Springer

[bib27] Matsuura K, Shiga K, Yokoyama J, Saijo S, Miyagi T, Takasaka T (1998) Loss of heterozygosity of chromosome 9p21 and 7q31 is correlated with high incidence of recurrent tumor in head and neck squamous cell carcinoma. Anticancer Res 18: 453–458 9568119

[bib28] Nawroz H, van der Riet, Hruban RH, Koch WM, Ruppert JM, Sidransky D (1994) Allelotype of head and neck squamous cell carcinoma. Cancer Res 54: 1152–1155 8118797

[bib29] Oba K, Matsuyama H, Yoshihiro S, Kishi F, Takahashi M., Tsukamoto M., Kinjo M., Sagiyama K, Naito K (2001) Two putative tumor suppressor genes on chromosome arm 8p may play different roles in prostate cancer. Cancer Genet Cytogenet 124: 20–26 11165318 10.1016/s0165-4608(00)00248-x

[bib30] Papadimitrakopoulou VA (2000) Carcinogenesis of head and neck cancer and the role of chemoprevention in its reversal. Curr Opin Oncol 12: 240–245 10841196 10.1097/00001622-200005000-00009

[bib31] Ransom DT, Barnett TC, Bot J, de Boer B, Mercalf C, Davidson JA, Turbett GR (1998) Loss of heterozygosity on chromosome 2q: possibly a poor prognostic factor in head and neck cancer. Head Neck 20: 404–410 9663668 10.1002/(sici)1097-0347(199808)20:5<404::aid-hed8>3.0.co;2-1

[bib32] Rainho CA, Kowalski LP, Rogatto SR (2001) Loss of imprinting and loss of heterozygosity on 11p15.5 in head and neck squamous cell carcinomas. Head Neck 23: 851–859 11592232 10.1002/hed.1124

[bib34] Renan MJ (1993) How many mutations are required for tumorigenesis? Implications from human cancer data. Mol Carcinogen 7: 139–146 10.1002/mc.2940070303 8489711

[bib35] Sasiadek M, Stembalska-Kozlowska A, Smigiel R, Schlade-Bartusiak K, Kupka S, Jagielski J, Krecicki T, Ramsey D, Mirghomizadeh F, Blin N (2002) Genetic instability in cancer: microsatellite and chromosomal instability in larynx squamous cell carcinoma. Int J Mol Med, (Suppl 1): 39

[bib37] Scholnick SB, Haughey BH, Sunwoo JB, El-Mofty SK, Baty JD, Piccirillo JF, Zequeira MR (1996) Chromosome 8 allelic loss and the outcome of patients with squamous cell carcinoma of the supraglottic larynx. J Natl Cancer Inst 88: 1676–1681 8931613 10.1093/jnci/88.22.1676

[bib36] Seifert M, Thesinger B, Engel M, Seib T, Seiz T, Stolte M, Hilgert K, Welter C (1997) Isolation and characterization of new microsatellites at the nm23-H1 and nm23-H2 gene loci and application for loss of heterozygosity (LOH) analysis. Hum Genet 100: 515–519 9341864 10.1007/s004390050545

[bib38] Sherr CJ (1996) Cancer cell cycles. Sciences 274: 1672–1677 10.1126/science.274.5293.1672 8939849

[bib39] Soder AI, Hopman AHN, Ramaekers FCS, Conradt C, Bosch FX (1995) Distinct nonrandom patterns of chromosomal aberrations in the progression of squamous cell carcinomas of the head and neck. Cancer Res 55: 5030–5037 7585547

[bib40] Sturgis EM, Wei Q (2002) Genetic susceptibility-molecular epidemiology of head and neck cancer. Curr Opin Oncol 14: 310–317 11981277 10.1097/00001622-200205000-00010

[bib41] Tsuda H, Takarabe T, Okada S, Uchida H, Kasamatsu T, Yamada Tsunematsu R, Ohmi K, Hirohashi S (2002) Different pattern of loss of heterozygosity among endocervicaltype adenocarcinoma, endometrioid-type adenocarcinoma and adenoma malignum of uterine cervix. Int J Cancer 98: 713–717 11920640 10.1002/ijc.10228

[bib42] Van Dyke DL, Worsham MJ, Benninger MS, Krause CJ, Baker SR, Wolf GT, Drumheller T, Tilley BC, Carey TE (1994) Recurrent cytogenetic abnormalities in squamous cell carcinomas of the head and neck region. Genes Chromosomes Cancer 9: 192–206 7515662 10.1002/gcc.2870090308

[bib43] Voravud N, Charurukus N, Mutirangura A (1997) Squamous cell carcinoma of head and neck. J Med Assoc Thai 80: 207–218 9175390

[bib44] Wheeler JMD, Beck NE, Kim HC, Tomlinson IPM, Mortensen NJMcC (1999) Mechanisms of inactivation of mismatch repair genes in human colorectal cancer cell lines: the prominent role of lMLH1. Proc Natl Acad Sci USA 96: 10296–10301 10468602 10.1073/pnas.96.18.10296 PMC17882

[bib45] Yokoyama J, Shiga K, Sasano H, Suzuki M, Takasaka T (1996) Abnormalities and the implication of retinoblastoma locus and its protein product in head and neck cancers. Anticancer Res 16: 641–644 8687109

